# Impact of preexisting interstitial lung disease on mortality in COVID-19 patients from the early pandemic to the delta variant epidemic: a nationwide population-based study

**DOI:** 10.1186/s12931-024-02723-3

**Published:** 2024-02-21

**Authors:** Koichi Miyashita, Hironao Hozumi, Kazuki Furuhashi, Eiji Nakatani, Yusuke Inoue, Hideki Yasui, Yuzo Suzuki, Masato Karayama, Noriyuki Enomoto, Tomoyuki Fujisawa, Naoki Inui, Toshiyuki Ojima, Takafumi Suda

**Affiliations:** 1 Second Division, Department of Internal Medicine, Hamamatsu University School of Medicine, 1-20-1 Handayama Higashiku, Hamamatsu, 431-3192 Japan; 2 Graduate School of Public Health, Shizuoka Graduate University of Public Health, 4-27-2 Kita Ando, Aoiku, 420-0881 Shizuoka Japan; 3 Department of Clinical Pharmacology and Therapeutics, Hamamatsu University School of Medicine, 1-20-1 Handayama, Hamamatsu, 431-3192 Japan; 4 Department of Community Health and Preventive Medicine, Hamamatsu University School of Medicine, 1-20-1 Handayama, Hamamatsu, 431-3192 Japan

**Keywords:** COVID-19, Mortality, Interstitial lung disease, National database of health insurance claims and specific health checkups of Japan, NDB

## Abstract

**Background:**

COVID-19 patients with preexisting interstitial lung disease (ILD) were reported to have a high mortality rate; however, this was based on data from the early stages of the pandemic. It is uncertain how their mortality rates have changed with the emergence of new variants of concern as well as the development of COVID-19 vaccines and treatments. It is also unclear whether having ILD still poses a risk factor for mortality. As COVID-19 continues to be a major concern, further research on COVID-19 patients with preexisting ILD is necessary.

**Methods:**

We extracted data on COVID-19 patients between January 2020–August 2021 from a Japanese nationwide insurance claims database and divided them into those with and without preexisting ILD. We investigated all-cause mortality of COVID-19 patients with preexisting ILD in wild-type-, alpha-, and delta-predominant waves, to determine whether preexisting ILD was associated with increased mortality.

**Results:**

Of the 937,758 adult COVID-19 patients, 7,333 (0.8%) had preexisting ILD. The proportion of all COVID-19 patients who had preexisting ILD in the wild-type-, alpha-, and delta-predominant waves was 1.2%, 0.8%, and 0.3%, respectively, and their 60-day mortality was 16.0%, 14.6%, and 7.5%, respectively. The 60-day mortality significantly decreased from the alpha-predominant to delta-predominant waves (difference − 7.1%, 95% confidence intervals (CI) − 9.3% to − 4.9%). In multivariable analysis, preexisting ILD was independently associated with increased mortality in all waves with the wild-type-predominant, odds ratio (OR) 2.10, 95% CI 1.91–2.30, the alpha-predominant wave, OR 2.14, 95% CI 1.84–2.50, and the delta-predominant wave, OR 2.10, 95%CI 1.66–2.66.

**Conclusions:**

All-cause mortality rates for COVID-19 patients with preexisting ILD decreased from the wild-type- to the more recent delta-predominant waves. However, these patients were consistently at higher mortality risk than those without preexisting ILD. We emphasize that careful attention should be given to patients with preexisting ILD despite the change in the COVID-19 environment.

**Supplementary Information:**

The online version contains supplementary material available at 10.1186/s12931-024-02723-3.

## Introduction

Coronavirus Disease 2019 (COVID-19) is an infectious disease caused by the severe acute respiratory syndrome coronavirus 2 (SARS-CoV-2). By May 2023, 760 million people had been infected with COVID-19 globally, with 6.9 million deaths [1]. Over this period, variants such as alpha, beta, gamma, delta, and omicron emerged, which the World Health Organization (WHO) designated as variants of concern (VOCs) [2]. Furthermore, the COVID-19 environment is changing due to the dissemination of vaccines and the development of therapeutic agents. We have reported previously that the clinical characteristics of COVID-19 patients changed, with decreasing mortality from the early pandemic to the delta variant epidemic [3]. We must continue to update the relevant evidence to improve COVID-19 management.

Preexisting interstitial lung disease (ILD) is a risk factor for mortality in COVID-19 patients, which is reported to range from 12 to 49% [4–9], and is higher than in patients without ILD [4–6]. However, these studies were conducted in the early pandemic, and it is unclear how the mortality of COVID-19 patients with preexisting ILD has changed since VOCs became prevalent and vaccines and COVID-19 therapies became available. Given that COVID-19 is still a major problem, further research on COVID-19 patients with preexisting ILD is needed.

The National Database of Health Insurance Claims and Specific Health Checkups of Japan (NDB) is one of the biggest medical databases in the world, covering most Japanese claims data [10]. We used this database to investigate the changes in the clinical characteristics and all-cause mortality of COVID-19 patients with preexisting ILD from the early pandemic to the delta variant epidemic. We also sought to clarify whether preexisting ILD posed an increased risk of all-cause mortality after COVID-19 diagnosis during each epidemic.

## Methods

### Dataset and waves

The NDB covers > 126 million people and 1.9 billion claims annually, including > 99% of Japanese inpatients and outpatients claims data [10]. This database contains information on age, sex, diseases based on the International Statistical Classification of Diseases and Related Health Problems, 10th revision (ICD-10), prescribed drugs and medical procedures covered by insurance, and mortality. It does not include information on smoking history, vaccinations, laboratory/physiological findings, and drugs not covered by insurance. We extracted anonymized information on adult patients with a confirmed diagnosis of COVID-19 between January 2020 and August 2021. During this period, the definitive diagnosis of COVID-19 in Japan was made mainly through nucleic acid amplification (e.g., reverse-transcription polymerase chain reaction) or antigen testing. In this study, COVID-19 patients were divided into those who already had underlying ILD before the onset of COVID-19 (preexisting ILD group) and those who did not (non-ILD group). The ICD-10 codes for any ILD, regardless of etiology, and for prespecified ILDs for this study (such as idiopathic pulmonary fibrosis [IPF], rheumatoid arthritis-associated ILD [RA-ILD], systemic lupus erythematosus-associated ILD [SLE-ILD], pulmonary sarcoidosis, etc. [11]). are listed in Additional file: Table S1. For patients’ pre-COVID-19 diagnosis comorbidities, information on cerebrovascular disease [12], malignancy [13], renal disease [14], congestive heart failure [12], liver disease [15], and diabetes mellitus [16] was extracted (Additional file: Table S2), with information on the use of long term oxygen therapy (LTOT) before COVID-19 diagnosis. For COVID-19 treatment, information on drugs, including corticosteroids, tocilizumab, baricitinib, heparin, and respiratory supportive care within 60 days of COVID-19 diagnosis, including oxygen therapy, high-flow nasal cannula, mechanical ventilation, and extracorporeal membrane oxygenation, was extracted. Death was defined as all-cause death within 60 days of COVID-19 diagnosis.

This database does not include information on the SARS-CoV-2 variants confirmed in each patient. As mentioned in our previous reports [3] based on the survey of the variants detected in Tokyo, Japan [17], when the detection rate of a VOC exceeded 50% of the tests performed, it was defined as the predominant VOC. The waves of the study period were (1) wild-type-predominant, from January 01, 2020 to April 18, 2021; (2) alpha-predominant, from April 19, 2021 to July 18, 2021; and (3) delta-predominant, from July 19, 2021 to August 31, 2021.

### Statistical analysis

Categorical variables are expressed as number (%). To compare proportions between waves, the differences and corresponding 95% confidence intervals (CI) were calculated using the Wald-test based method. We used multivariate logistic regression models adjusted for age, sex, with/without wave, and comorbidities to explore the association of preexisting ILD with all-cause mortality. The odds ratio (OR) and 95% CI were also calculated. The multicollinearity between variables was checked. A *P*-value of < 0.05 was considered statistically significant. However, due to the large sample size in this study, absolute standardized differences (ASDs) were presented to enable us to assess differences in the baseline characteristic variables between two groups. When the ASD was < 0.1, the variables between the two groups were taken as approximately equivalent, even if the *P*-value was significant. All data were analyzed using SAS software, version 9.4 (SAS Institute Inc., NC, USA).

## Results

### Patient characteristic and mortality

A total of 937,758 adult COVID-19 patients were identified. Of these, 7,333 (0.8%) had preexisting ILD and 930,425 (99.2%) did not. The clinical characteristics of the groups are shown in Table 1. Patients in the preexisting ILD group were significantly older than those in the non-ILD group (median age category; 70–74 years and 40–44 years, respectively; ASD 1.52). The proportion of patients who had received LTOT before the COVID-19 diagnosis was also higher in the preexisting ILD group than in the non-ILD group (7.7% and 0.2%, respectively; ASD 0.40).

**Table 1:** Patient characteristics

	preexisting ILD *n* = 7,333	non-ILD *n* = 930,425	Absolute standardized difference
Age, years	70–74^d^	40–44^d^	1.52
20–49	629 (8.6)	576,627 (62.0)	
50–64	1,249 (17.0)	183,839 (19.8)	
65–79	3,063 (41.8)	101,498 (10.9)	
80+	2,392 (32.6)	68,461 (7.4)	
Sex, male	4,181 (57.0)	506,854 (54.5)	0.05
Comorbidity:			
Cerebrovascular disease	1,746 (23.8)	52,304 (5.6)	0.53
Malignancy	2,354 (32.1)	40,500 (4.4)	0.77
Renal disease	1,052 (14.3)	19,586 (2.1)	0.46
Congestive heart failure	3,042 (41.5)	54,733 (5.9)	0.92
Liver disease	1963 (26.8)	75,638 (8.1)	0.51
Diabetes mellitus	3,962 (54.0)	108,140 (11.6)	1.01
LTOT before COVID-19 diagnosis	568 (7.7)	1,426 (0.2)	0.40
ILD type:			
Idiopathic pulmonary fibrosis	295 (4.0)		
RA-associated ILD	1,795 (24.5)		
SLE-associated ILD	427 (5.8)		
PM/DM-associated ILD	333 (4.5)		
SSc-associated ILD	293 (4.0)		
SjS-associated ILD	493 (6.7)		
MPA-associated ILD	138 (1.9)		
Pulmonary sarcoidosis	231 (3.2)		
Pneumoconiosis	213 (2.9)		
Hypersensitivity pneumonitis	69 (0.9)		
Other ILD^a^	4,220 (57.6)		
COVID-19 treatment:			
Corticosteroids^b^	3,040 (41.5)	134,139 (14.4)	0.63
Steroid pulse^c^	709 (9.7)	11,657 (1.3)	0.38
Tocilizumab	276 (3.8)	7,447 (0.8)	0.20
Baricitinib	169 (2.3)	11,778 (1.3)	0.08
Heparin	1,127 (15.4)	36,500 (3.9)	0.40
Respiratory support care:			
Oxygen therapy	3,238 (44.2)	97,986 (10.5)	0.81
High-flow nasal cannula	439 (6.0)	10,401 (1.1)	0.27
Mechanical ventilation	505 (6.9)	13,096 (1.4)	0.28
ECMO	21 (0.3)	778 (0.1)	0.05
60-day mortality	1,039 (14.2)	15,665 (1.7)	0.48

Data are presented as median age category or number (%)

^a^ Langerhans cell histiocytosis, lymphangioleiomyomatosis, radiation pneumonitis, eosinophilic pneumonia, granulomatosis with polyangiitis-associated ILD, eosinophilic granulomatosis with polyangiitis-associated ILD, mixed connective tissue disease-associated ILD, idiopathic interstitial pneumonias other than idiopathic pulmonary fibrosis, and unspecified ILD

^b^ Corticosteroids newly administered within 60 days of COVID-19 diagnosis or corticosteroid dosage increased within 60 days of COVID-19 diagnosis in patients on corticosteroids before diagnosis

^c^ The use of corticosteroids equivalent to 500 mg or more of methylprednisolone at least once within 60 days of COVID-19 diagnosis

^d^ Median age category

DM, dermatomyositis; ILD, interstitial lung disease; ECMO, extracorporeal membrane oxygenation; LTOT, long term oxygen therapy; MPA, microscopic polyangiitis; PM, polymyositis; RA, rheumatoid arthritis; SjS, Sjogren syndrome; SLE, systemic lupus erythematosus; SSc, systemic sclerosis

The proportion of patients who were treated with corticosteroids was higher in the preexisting ILD group than in the non-ILD group (41.5% and 14.4%, respectively; ASD 0.63). A higher proportion of patients in the preexisting ILD group than in the non-ILD group received oxygen therapy (44.2% and 10.5%, respectively; ASD 0.81), high-flow nasal cannula (HFNC) (6.0% and 1.1%, respectively; ASD 0.27), and mechanical ventilation (6.9% and 1.4%, respectively; ASD 0.28). The 60-day mortality was higher in the preexisting ILD group than in the non-ILD group (14.2% and 1.7%, respectively, ASD 0.48).

### Patient characteristics and outcome by etiology of preexisting ILD

Patient characteristics by etiology of preexisting ILD are shown in Additional file: Table S3. The number of patients who died within 60 days of COVID-19 diagnosis was 64 of 295 (21.7%) patients with IPF, 177 of 1,795 (9.9%) with rheumatoid arthritis-associated ILD (RA-ILD), 34 of 427 (8.0%) with systemic lupus erythematosus-associated ILD (SLE-ILD), 30 of 333 (9.0%) with polymyositis/dermatomyositis-associated ILD, 26 of 293 (8.9%) with systemic sclerosis-associated ILD (SSc-ILD), 33 of 493 (6.7%) with Sjogren syndrome-associated ILD, 22 of 138 (15.9%) with Microscopic polyangiitis-associated ILD, 12 of 231 (5.2%) with pulmonary sarcoidosis, 42 of 213 (19.7%) with pneumoconiosis, and < 10 of 69 (< 14.5%) with hypersensitivity pneumonitis.

### Patient characteristics and mortality by waves

**Fig. 1:**
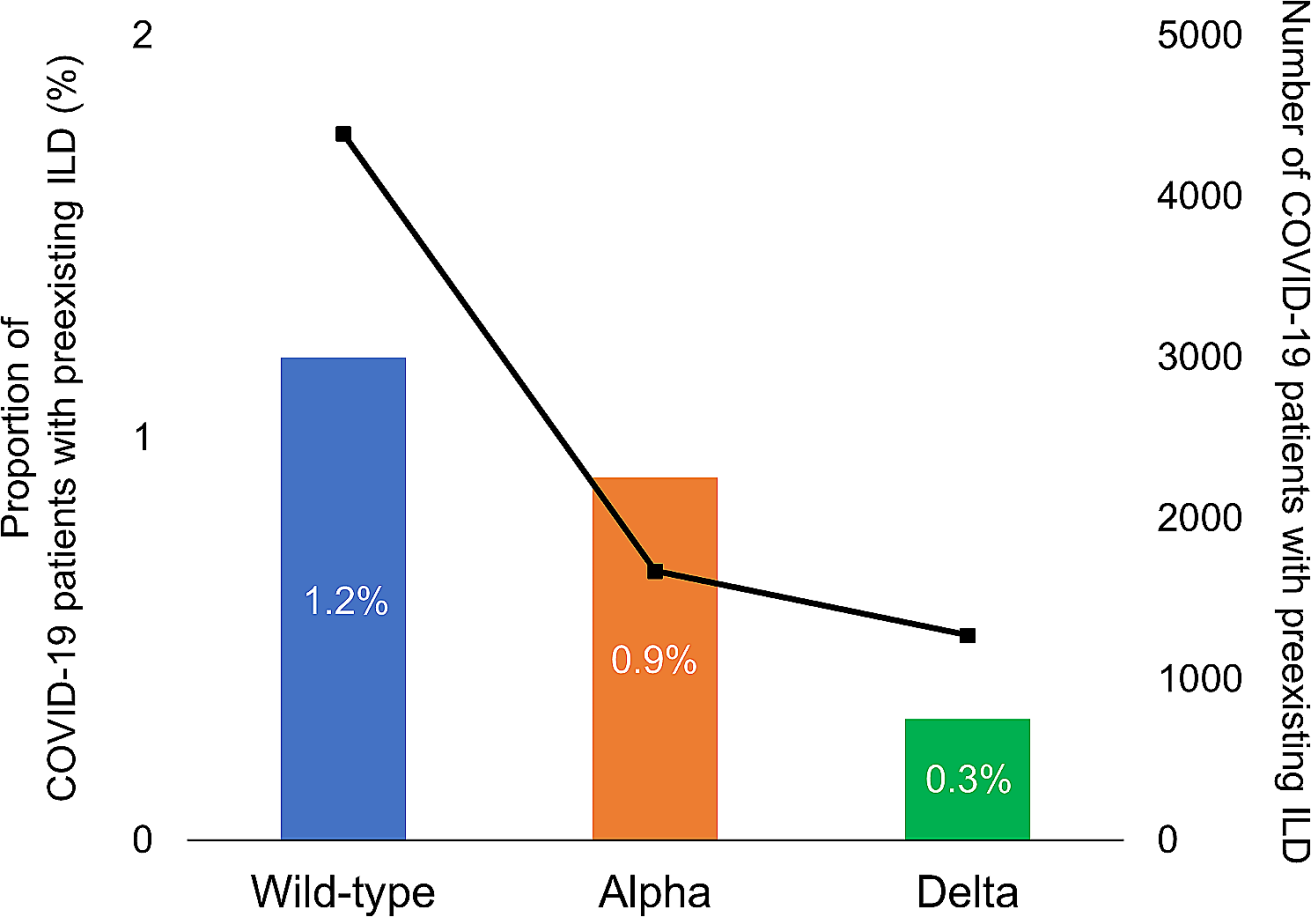
Proportion of COVID-19 patients with preexisting interstitial lung disease by wave. Among total COVID-19 patients in each wave, the proportion of COVID-19 patients with preexisting interstitial lung disease was 1.2% (4387/365,929), 0.8% (1673/196,957) and 0.3% (1273/374,872), in the wild-type-, alpha-, and delta-predominant wave, respectively. Wild-type-predominant wave, January 01, 2020–April 18, 2021; alpha-predominant wave, April 19, 2021–July 18, 2021; delta-predominant wave, July 19, 2021–August 31, 2021

In the preexisting ILD group, fewer patients were diagnosed with COVID-19 as the waves shifted (Table 2). The proportion of patients with preexisting ILD among all COVID-19 patients in each wave also decreased from the wild-type-predominant to the alpha-predominant waves (1.2% and 0.8%, respectively; difference − 0.4%, 95% CI − 0.4% to − 0.3%) and from the alpha-predominant to the delta-predominant waves (0.8% and 0.3%, respectively; difference − 0.5%, 95% CI − 0.6% to − 0.5%) (Fig. 1). As the wave shifted, the number of patients aged ≥ 65 years and their proportion of all patients with ILD decreased markedly from the alpha-predominant to the delta-predominant waves (74.8–56.8%, respectively; difference − 18.2%, 95%CI − 21.6% to − 14.8%) (Table 2). A similar decrease was observed in the non-ILD group (Additional file: Figs. S1 and S2).

**Table 2:** Characteristics of COVID-19 patients with preexisting interstitial lung disease by wave

		Waves^a^			Wild-type vs. Alpha^b^	Alpha vs. Delta^b^	Wild-type vs. Delta^b^
	Wild-type *n* = 4,387	Alpha *n* = 1,673	Delta *n* = 1,273		Difference % (95% CI)	Difference % (95% CI)	Difference % (95% CI)
Age, years:	75–79^e^	70–74^e^	65–69^e^				
20–49	274 (6.2)	129 (7.7)	226 (17.8)		1.5 (0 to 2.9)	10.0 (7.6 to 12.5)	11.5 (9.3 to 13.7)
50–64	631 (14.4)	292 (17.5)	326 (25.6)		3.1 (1.0 to 5.2)	8.2 (5.1 to 11.2)	11.2 (8.6 to 13.8)
65–79	1931 (44.0)	709 (42.4)	423 (33.2)		−1.6 (− 4.4 to 1.1)	−9.2 (− 12.7 to − 5.6)	−10.8 (− 13.8 to − 7.8)
80+	1,551 (35.4)	543 (32.5)	298 (23.4)		−2.9 (− 5.6 to − 0.2)	−9.0 (− 12.3 to − 5.8)	−11.9 (− 14.7 to − 9.2)
Sex, male	2,558 (58.3)	962 (57.5)	661 (51.9)		−0.8 (− 3.6 to 2.0)	−5.6 (− 9.2 to − 2.0)	−6.4 (− 9.5 to − 3.3)
Comorbidity:							
Cerebrovascular disease	1,133 (25.8)	394 (23.6)	219 (17.2)		−2.3 (− 4.7 to 0.1)	−6.3 (− 9.3 to − 3.4)	−8.6 (− 11.1 to − 6.2)
Malignancy	1,482 (33.8)	508 (30.4)	364 (28.6)		−3.4 (− 6.0 to − 0.8)	−1.8 (− 5.1 to 1.5)	−5.2 (− 8.0 to − 2.3)
Renal disease	662 (15.1)	221 (13.2)	169 (13.3)		−1.9 (− 3.8 to 0.1)	0.1 (− 2.4 to 2.5)	−1.8 (− 4.0 to 0.3)
Congestive heart failure	1906 (43.4)	681 (40.7)	455 (35.7)		−2.7 (− 5.5 to 0)	−5.0 (− 8.5 to − 1.4)	−7.7 (− 10.7 to − 4.7)
Liver disease	1,210 (27.6)	445 (26.6)	308 (24.2)		−1.0 (− 3.5 to 1.5)	−2.4 (− 5.6 to 0.8)	−3.4 (− 6.1 to − 0.7)
Diabetes mellitus	2,415 (55.0)	896 (53.6)	651 (51.1)		−1.5 (− 4.3 to 1.3)	−2.4 (− 6.1 to 1.2)	−3.9 (− 7.0 to − 0.8)
LTOT before COVID-19 diagnosis	383 (8.7)	106 (6.3)	79 (6.2)		−2.4 (− 3.8 to − 1.0)	−0.1 (− 1.9 to 1.6)	−2.5 (− 4.1 to − 1.0)
Treatment for COVID-19:							
Corticosteroids^c^	1,894 (43.2)	752 (45.0)	394 (31.0)		1.8 (− 1.0 to 4.6)	−14.0 (− 17.5 to − 10.5)	−12.2 (− 15.2 to − 9.3)
Steroid pulse^d^	480 (10.9)	169 (10.1)	60 (4.7)		−0.8 (− 2.6 to 0.9)	−5.4 (− 7.2 to − 3.5)	−6.2 (− 7.7 to − 4.7)
Tocilizumab	150 (3.4)	79 (4.7)	47 (3.7)		1.3 (0.2 to 2.5)	−1.0 (− 2.5 to 0.4)	0.3 (− 0.9 to 1.4)
Baricitinib	22 (0.5)	96 (5.7)	51 (4.0)		5.2 (4.1 to 6.4)	−1.7 (− 3.3 to − 0.2)	3.5 (2.4 to 4.6)
Heparin	747 (17.0)	266 (15.9)	114 (9.0)		−1.1 (− 3.2 to 0.9)	−6.9 (− 9.3 to − 4.6)	−8.1 (− 10.0 to − 6.1)
Respiratory support care:							
Oxygen therapy	2,116 (48.2)	748 (44.7)	374 (29.4)		−3.5 (− 6.3 to − 0.7)	−15.3 (− 18.8 to − 11.9)	−18.9 (− 21.8 to − 15.9)
High-flow nasal cannula	248 (5.7)	134 (8.0)	57 (4.5)		2.4 (0.9 to 3.8)	−3.5 (− 5.3 to − 1.8)	−1.2 (− 2.5 to 0.2)
Mechanical ventilation	379 (8.6)	103 (6.2)	23 (1.8)		−2.5 (− 3.9 to − 1.1)	−4.4 (− 5.7 to − 3.0)	−6.8 (− 7.9 to − 5.7)
60-day mortality	700 (16.0)	244 (14.6)	95 (7.5)		−1.4 (− 3.4 to 0.6)	−7.1 (− 9.3 to − 4.9)	−8.5 (− 10.3 to − 6.7)

Data are presented as number (%)

^a^ Wild-type-predominant wave, January 01, 2020–April 18, 2021; alpha-predominant wave, April 19, 2021–July 18, 2021; delta-predominant wave, July 19, 2021–August 31, 2021

^b^ Earlier wave was used as reference

^c^ Corticosteroids newly administered within 60 days of COVID-19 diagnosis or corticosteroid dosage increased within 60 days of diagnosis in patients who had been using corticosteroids prior to COVID-19 diagnosis

^d^ Corticosteroid use equivalent to 500 mg or more of methylprednisolone at least once within 60 days of COVID-19 diagnosis

^e^ Median age category

CI, confidence interval; LTOT, long term oxygen therapy

**Fig. 2:**
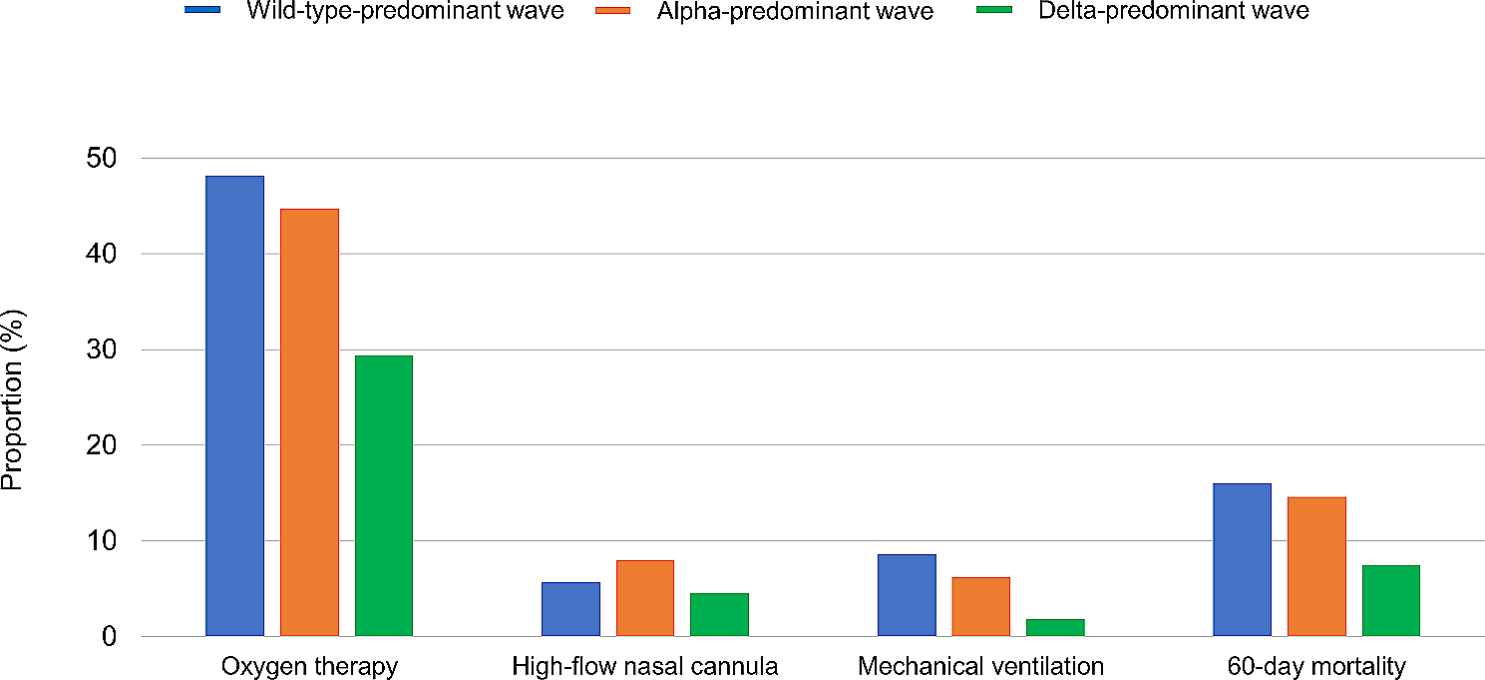
Respiratory support care and mortality in COVID-19 patients with preexisting interstitial lung disease by wave. Among the COVID-19 patients with preexisting interstitial lung disease in each wave, the proportion of patients who required oxygen therapy were 48.2%, 44.7%, and 29.4% in the wild-type-, alpha-, and delta-predominant wave, respectively. The proportion of patients who required high-flow nasal cannula was 5.7%, 8.0%, and 4.5% in the wild-type-, alpha-, and delta-predominant wave, respectively. The proportion of patients who required mechanical ventilation was 8.6%, 6.2%, and 1.8% in the wild-type-, alpha-, and delta-predominant wave, respectively. The proportion of death in COVID-19 patients with interstitial lung disease was 16.0%, 14.6%, and 7.5%, in wild-type-, alpha-predominant wave, delta-predominant wave, respectively. Wild-type-predominant wave, January 01, 2020–April 18, 2021; alpha-predominant wave, April 19, 2021–July 18, 2021; delta-predominant wave, July 19, 2021–August 31, 2021

Changes in respiratory supportive care and mortality are shown in Fig. 2 and Table 2. As the wave shifted from the wild-type-, to alpha-, and delta-predominant waves, the proportions of patients receiving oxygen therapy and mechanical ventilation decreased. The 60-day mortality rates in the wild-type-, alpha-, and delta-predominant waves were 16.0%, 14.6%, and 7.5%, respectively. The mortality rates decreased significantly from the alpha-predominant to the delta-predominant waves (difference − 7.1%, 95% CI − 9.3% to − 4.9%). There was also a decrease in the use of respiratory support care and 60-day mortality in the non-ILD group (Additional file: Table S4).

### Change of mortality by etiology in patients with preexisting interstitial lung disease

The 60-day mortality rates by etiology in patients with ILD are shown in Additional file: Table S5. COVID-19 patients with preexisting IPF had the highest 60-day mortality rate in each wave; 23.6%, 19.0%, and 16.7% for the wild-type, alpha-, and delta-predominant wave, respectively, with no significant decrease in mortality from the wild-type predominant to the alpha-predominant waves (difference − 4.6%, 95% CI − 16.3–7.1%), from the alpha-predominant to the delta-predominant waves (difference − 2.3%, 95% CI − 17.4–12.8%), and from the wild-type predominant to the delta-predominant waves (difference − 6.9%, 95% CI − 19.7–5.8%). The 60-day mortality decreased significantly from the alpha- to the delta-predominant waves in RA-ILD, difference − 5.6% (95% CI − 9.1% to − 2.0%), SLE-ILD − 6.7% (95% CI − 12.7% to − 0.6%), and pulmonary sarcoidosis, − 8.9% (95% CI − 17.5% to − 0.4%).

### Association of preexisting interstitial lung disease and mortality in COVID-19 patients

**Fig. 3:**
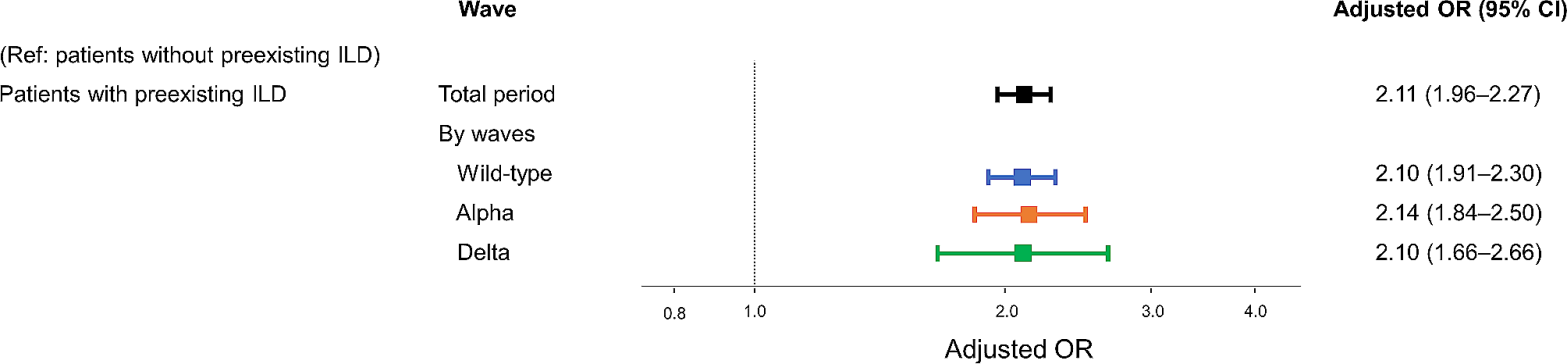
The impact of preexisting interstitial lung disease on mortality in COVID-19 patients in the total period and for each wave. Adjusted odds ratios and 95% confidence intervals were plotted. Multivariate logistic regression models adjusted for age, sex, wave, and comorbidities in the total period, and adjusted for age, sex, and comorbidities in each wave. Total period, January 01, 2020–August 31, 2021; wild-type-predominant wave, January 01, 2020–April 18, 2021; alpha-predominant wave, April 19, 2021–July 18, 2021; delta-predominant wave, July 19, 2021–August 31, 2021. CI, confidence interval; ILD, interstitial lung disease; OR, odds ratio

In the multivariate logistic regression model, preexisting ILD was independently associated with increased mortality over the total period (OR 2.11, 95% CI 1.96–2.27) (Fig. 3). In all waves, preexisting ILD was consistently associated with increased mortality; in the wild-type-predominant wave (OR 2.10, 95% CI 1.91–2.30), in the alpha-predominant wave (OR 2.14, 95% CI 1.84–2.50), and in the delta-predominant wave (OR 2.10, 95% CI 1.66–2.66).

**Fig. 4:**
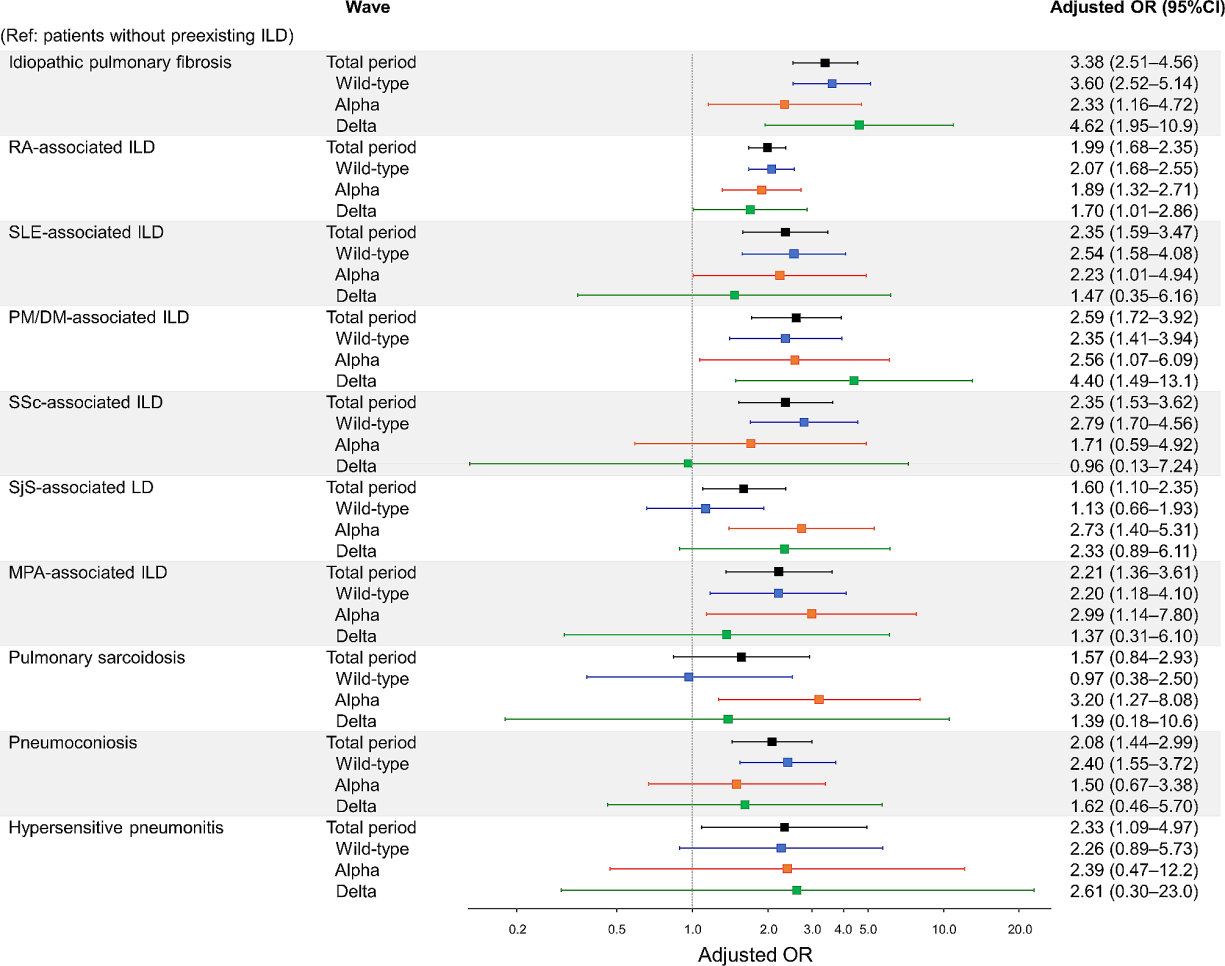
The impact of types of preexisting interstitial lung disease on mortality in COVID-19 patients in the total period and each wave. Adjusted odds ratios and 95% confidence intervals were plotted. Multivariate logistic regression models adjusted for age, sex, wave, and comorbidities in the total period, and adjusted for age, sex, and comorbidities in each wave. Total period, January 01, 2020–August 31, 2021; wild-type-predominant wave, January 01, 2020–April 18, 2021; alpha-predominant wave, April 19, 2021–July 18, 2021; delta-predominant wave, July 19, 2021–August 31, 2021. CI, confidence interval; DM, dermatomyositis; ILD, interstitial lung disease; MPA, microscopic polyangiitis; OR, odds ratio; PM, polymyositis; RA, rheumatoid arthritis; SjS, Sjogren syndrome; SLE, systemic lupus erythematosus; SSc, systemic sclerosis

The results of the multivariate analysis by etiology of preexisting ILD are shown in Fig. 4. Over the total period, preexisting ILD of all etiologies was consistently associated with increased mortality. The OR was particularly high in IPF (OR 3.38, 95% CI 2.51–4.56) relative to ILD of other etiologies. In subgroup analyses by wave, ILD of all etiologies except SSc-ILD and pulmonary sarcoidosis were consistently associated with increased mortality.

## Discussion

This is the first study to investigate the changes in characteristics and all-cause mortality of patients with COVID-19 who had underlying ILD from the early pandemic to the delta variant epidemic using a large-scale database. As the waves evolved, the number of patients with preexisting ILD and their proportion among all patients with COVID-19 decreased. In the preexisting ILD group, the number and proportion of elderly patients, and patients who required oxygen therapy, HFNC, and mechanical ventilation also decreased. Furthermore, the number of deaths and all-cause mortality rates within 60 days of COVID-19 diagnosis also decreased. However, in all waves, having preexisting ILD was consistently associated with a higher mortality than not having an ILD.

In this study, even as the waves shifted from the wild-type- to alpha-, and delta-predominant waves, the overall number of patients with COVID-19 remained high (approximately 360,000, 200,000, and 370,000, respectively), while the number of patients with preexisting ILD and their proportion among all patients decreased markedly. Since the COVID-19 vaccine was not widely available during the alpha-predominant wave in Japan, the decrease in the number and proportion of patients with preexisting ILD from the wild-type- to the alpha-predominant waves can be assumed to be mainly due to the patients’ efforts to prevent infection. For example, vulnerable patients at high risk of severe disease or death may have stayed indoors or practiced strict social distancing. However, the similar decrease from the alpha- to delta-predominant waves may also be due to widespread vaccination. In Japan, the vaccination program started in the middle of the alpha-dominant wave, giving priority to patients aged ≥ 65 years or with a comorbidity. During the delta-predominant wave, the second vaccination coverage was about 20% for those aged < 65 years and about 90% for those aged ≥ 65 years [18]. As shown in Table 2, from alpha- to delta-predominant waves, the number of patients with preexisting ILD aged < 65 years did not decrease, while the number of those aged ≥ 65 years decreased significantly.

The 30-day mortality rate for patients with COVID-19 and preexisting ILD was reported to be 25.2% in a study by Gallay et al. [7] and 13.4% between January and June 2020 in a Korean study using nationwide data [6]; both studies were based on data from the early pandemic period. The present study showed that the 60-day mortality rates in the wild-type and alpha-dominant waves were 16.0% and 14.6%, respectively. Although the delta variant was considered to be as virulent as the alpha variant [19], we found that in the delta-predominant wave, the number and proportion of patients requiring respiratory supportive care including oxygen therapy, HFNC, and mechanical ventilation, and the 60-day mortality rate (7.5%) decreased significantly in patients with preexisting ILD. Similar decreases were observed in the non-ILD group. The exact reason for this decline is not clear, but it may be associated with the availability of vaccines and the development of COVID-19 therapies. The number of infected elderly patients at high risk of severe disease or mortality, decreased during the period of high coverage of the second vaccination. In addition, even when infected, the vaccination may have reduced the risk of severe outcomes. Before the delta-predominant wave, therapeutic regimens with dexamethasone, baricitinib, and remdesivir were developed [20–23], and casirivimab/imdevimab became available in Japan during the delta-predominant wave [24, 25]. These improvements may have contributed to a decrease in mortality in patients with and without ILD.

Studies in the early pandemic period reported that COVID-19 patients who had preexisting ILD had a higher risk of mortality than those without ILD [4–6]. However, it is unclear whether this remained true during the VOC epidemics after the early pandemic period. This study found that although 60-day mortality in patients with preexisting ILD decreased as the wave shifted, having a preexisting ILD was consistently associated with increased mortality in the wild-type-, alpha- and delta-predominant waves. This suggests that regardless of changes in prevalent variants, widespread vaccination, and the development of treatments, patients with preexisting ILD are at high mortality risk for COVID-19, and we should be vigilant when managing these patients in clinical practice.

Among the etiologies of preexisting ILDs, the 60-day mortality rate of patients who had IPF was the highest in any wave. While the rate in patients with other ILDs, including RA-ILD, SLE-ILD, and pulmonary sarcoidosis, decreased significantly from the alpha- to delta-predominant waves, the 60-day mortality of patients with IPF did not decrease significantly. Furthermore, having IPF was independently associated with increased mortality in all waves, compared to those without preexisting ILD. Therefore, thorough preventive measures, including vaccination, should be taken by ILD patients and early and aggressive treatment should be initiated if infected, especially in patients with IPF.

This study had several limitations. First, the database does not include SARS-CoV-2 variant information for individual patients. Second, the NDB does not include vaccination history for each patient. Third, the inclusion criteria for patients with ILD were based on ICD-10 codes, thus a misclassification of the diagnosis of each ILD type might be present. Forth, the NDB does not include data on cause of death. As it was not feasible to distinguish whether deaths within 60 days of COVID-19 diagnosis in patients with preexisting ILD were due to COVID-19, ILD, or other causes, all-cause mortality was reported in this study. Fifth, the data were derived from patients diagnosed up to the delta-predominant wave, and further studies are required to understand the patterns of the omicron-predominant wave.

In conclusion, the clinical characteristics of COVID-19 in patients with preexisting ILD changed from the early pandemic to the delta-predominant wave, including a decrease in the 60-day mortality. However, compared to those without, COVID-19 patients with preexisting ILD were consistently at higher risk of all-cause mortality. We emphasize that careful attention should be given to patients with preexisting ILD despite the change in the COVID-19 environment.

### Electronic supplementary material

Below is the link to the electronic supplementary material.

Supplementary Material 1

## Data Availability

The data that support the findings of this study are available from the Japan Ministry of Health, Labor, and Welfare. Restrictions apply to the availability of these data, which were used under license for this study.

## Abbreviations

ASD: Absolute standardized difference
CI: Confidence interval
COVID-19: Coronavirus Disease 2019
ECMO: Extracorporeal membrane oxygenation
HFNC: High-flow nasal canula
HP: Hypersensitivity pneumonitis
ICD-10: International Statistical Classification of Diseases and Related Health Problems
ILD: Interstitial lung disease
IPF: Idiopathic pulmonary fibrosis
LTOT: Long term oxygen therapy
MPA-ILD: Microscopic polyangiitis-associated interstitial lung disease
NDB: National Database of Health Insurance Claims and Specific Health Checkups of Japan
OR: Odds ratio
PM/DM-ILD: Polymyositis/dermatomyositis-associated interstitial lung disease
RA-ILD: Rheumatoid arthritis-associated interstitial lung disease
SARS-CoV-2: Severe acute respiratory syndrome coronavirus 2
SjS-ILD: Sjogren syndrome-associated interstitial lung disease
SLE-ILD: Systemic lupus erythematosus-associated interstitial lung disease
SSc-ILD: Systemic sclerosis-associated interstitial lung disease
VOC: Variants of concern
WHO: World Health Organization
