# Supplementary material for: Impact of preexisting interstitial lung disease on mortality in COVID-19 patients from the early pandemic to the delta variant epidemic: a nationwide population-based study
Source: Respir Res. 2024 Feb 21;25:95. doi: 10.1186/s12931-024-02723-3 (PMC10880313; doi:10.1186/s12931-024-02723-3)
Supplement: Supplementary file 1 — Supplementary Material 1 [file 12931_2024_2723_MOESM1_ESM.docx]

**Additional files**

**Fig. S1. Age distribution of COVID-19 patients with preexisting interstitial lung disease.**

**Fig. S2. Age distribution of COVID-19 patients without preexisting interstitial lung disease.**

**Table S1. List of ICD-10 codes for interstitial lung diseases.**

**Table S2. List of ICD-10 codes for comorbidities.**

**Table S3. Characteristics by disease in COVID-19 patients with preexisting interstitial lung disease.**

**Table S4. Characteristics of COVID-19 patients without preexisting interstitial lung disease by wave.**

**Table S5. Change of mortality by etiology in COVID-19 patients with preexisting interstitial lung disease.**

**Fig. S1. Age distribution of COVID-19 patients with preexisting interstitial lung disease.**


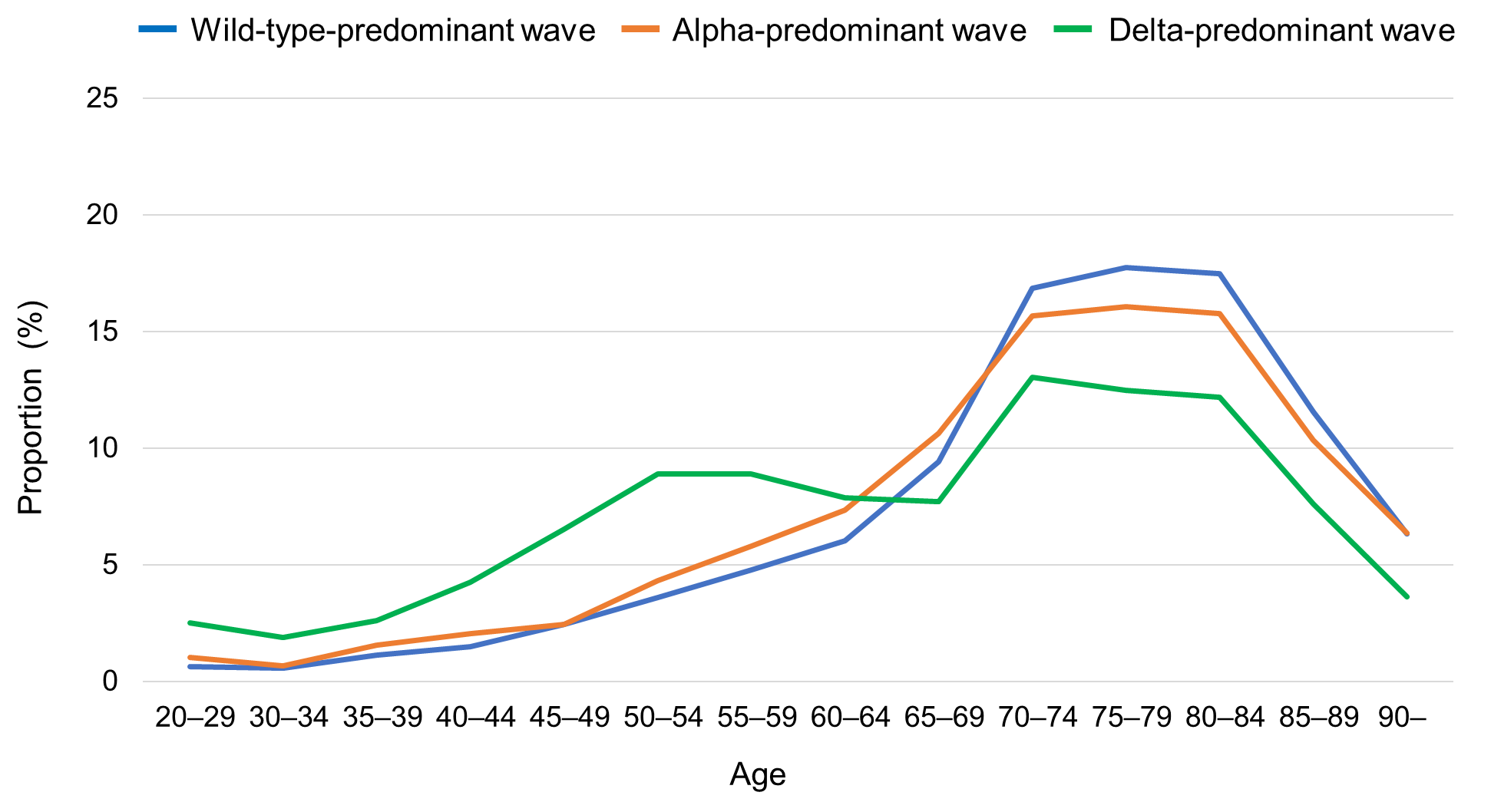


Wild-type-predominant wave, January 01, 2020–April 18, 2021; alpha-predominant wave, April 19, 2021–July 18, 2021; delta-predominant wave, July 19, 2021–August 31, 2021.

The guidelines of the Japanese Ministry of Health, Labor, and Welfare prohibit the publication of specific numbers of variables with fewer than 10 patients for anonymity reasons. Because the number of patients in 20–24 age category was fewer than 10, it was combined with the number of patients in 25–29 age category and reported as the 20–29 age category.

**Fig. S2. Age distribution of COVID-19 patients without preexisting interstitial lung disease.**


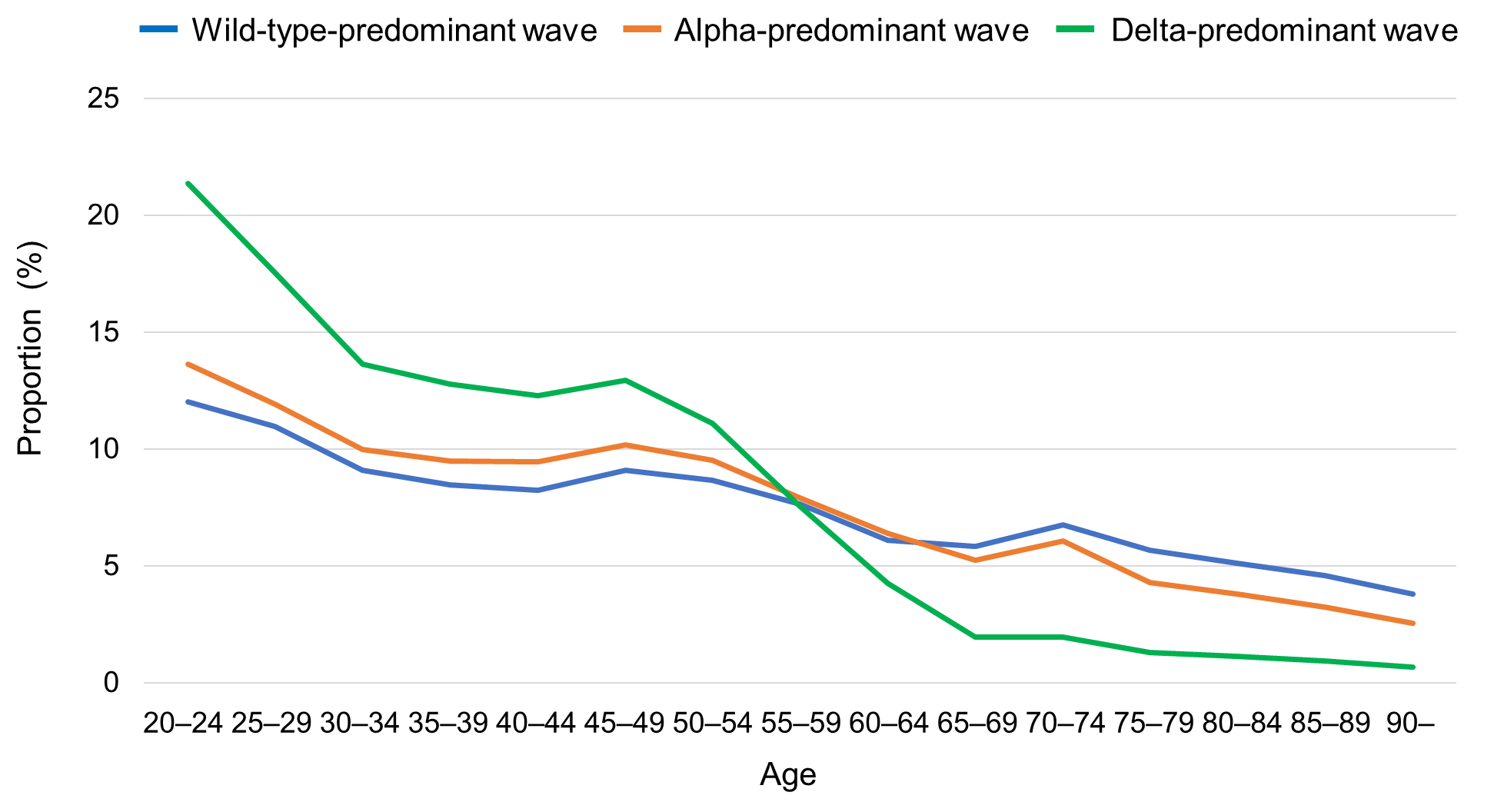


Wild-type-predominant wave, January 01, 2020–April 18, 2021; alpha-predominant wave, April 19, 2021–July 18, 2021; delta-predominant wave, July 19, 2021–August 31, 2021.

**Table S1.** **List of ICD-10 codes for interstitial lung diseases.**

| **Category** | **ICD-10 code** |
| --- | --- |
| **Any ILD** | C96.6, D21.9, D86.0, J60-64, J66-67, J70, J82, J84, J99.1 |
| **ILD type** |  |
| Idiopathic pulmonary fibrosis | J84.112 |
| Rheumatoid arthritis-associated ILD | M05–06 + J84.1–84.9 or J99.1 |
| Systemic lupus erythematosus-associated ILD | M32 + J84.1–84.9 or J99.1 |
| Polymyositis/dermatomyositis-associated ILD | M33 + J84.1–84.9 or J99.1 |
| Systemic sclerosis-associated ILD | M34 + J84.1–84.9 or J99.1 |
| Sjogren syndrome-associated ILD | M35.0 + J84.1–84.9 or J99.1 |
| Microscopic polyangiitis-associated ILD | M31.7 + J84.1–84.9 |
| Pulmonary sarcoidosis | D86 + J84.1–J84.9 or D86.0 |
| Pneumoconiosis | J60–64 |
| Hypersensitivity pneumonitis | J66–67 |
| Other ILDs ^a^ | C96.6, D21.9, J66–67, J70, J82, J84.0, J84.1 excluding J84.112, J84.9, M301, or M313 |

^a^ Langerhans cell histiocytosis, lymphangioleiomyomatosis, radiation pneumonitis, eosinophilic pneumonia, granulomatosis with polyangiitis-associated ILD, eosinophilic granulomatosis with polyangiitis-associated ILD, mixed connective tissue disease-associated ILD, idiopathic interstitial pneumonias other than idiopathic pulmonary fibrosis, unspecified ILD.

ILD, interstitial lung disease

**Table S2. List of ICD-10 codes for comorbidities.**

| **Category** | **ICD-10 code** |
| --- | --- |
| Cerebrovascular disease | G45–46, I60–69, H34.0 |
| Malignancy | C00–26, C30–34, C37–41, C43, C45–58, C60–76, C81–85, C88, C90–97 |
| Renal disease | N18–19, I12.0, I13.1, N03.2–N03.7, N05.2–N05.7, N25.0, Z49.0–49.2, Z94.0, Z99.2 |
| Congestive heart failure | I43, I50, I09.9, I11.0, I13.0, I13.2, I25.5, I42.0, I42.5–42.9, P29.0 |
| Liver disease | B18, I85.0, I85.9, I86.4, I98.2, K70.0–70.4, K70.9, K71.1, K71.3–71.5, K71.7, K72.1, K72.9, K76.0, K76.2–76.9, Z94.4 |
| Diabetes mellitus | E10–14 |

**Table S3. Characteristics by disease in COVID-19 patients with preexisting interstitial lung disease.**

|  | **IPF**  **n = 295** | **RA-ILD**  **n = 1,795** | **SLE-ILD**  **n = 427** | **PM/DM-ILD**  **n = 333** | **SSc-ILD**  **n = 293** | **SjS-ILD**  **n = 493** | **MPA-ILD**  **n = 138** | **Pulmonary sarcoidosis**  **n = 231** | **Pneumoconiosis**  **n = 213** | **Hypersensitivity pneumonitis**  **n = 69** |
| --- | --- | --- | --- | --- | --- | --- | --- | --- | --- | --- |
| Age, years: | 75–79^d^ | 70–74^d^ | 60–64^d^ | 65–69^d^ | 65–69^d^ | 65–69^d^ | 75–79^d^ | 60–64^d^ | 75–79^d^ | 70–74^d^ |
| 20–49 | 32 (10.8)^e^ | 208 (11.6) | 101 (23.7) | 60 (18.0) | 37 (12.6) | 82 (16.6) | 24 (17.4)^e^ | 65 (28.1) | 11 (5.2) | 18 (26.1)^e^ |
| 50–64 |  | 410 (22.8) | 131 (30.7) | 95 (28.5) | 78 (26.6) | 130 (26.4) |  | 64 (27.7) | 21 (9.9) |  |
| 65–79 | 162 (54.9) | 735 (40.9) | 130 (30.4) | 128 (38.4) | 120 (41.0) | 173 (35.1) | 66 (47.8) | 72 (31.2) | 85 (39.9) | 33 (47.8) |
| 80+ | 101 (34.2) | 442 (24.6) | 65 (15.2) | 50 (15.0) | 58 (19.8) | 108 (21.9) | 48 (34.8) | 30 (13.0) | 96 (45.1) | 18 (26.1) |
| Sex, male | 214 (72.5) | 636 (35.4) | 110 (25.8) | 128 (38.4) | 56 (19.1) | 91 (18.5) | 64 (46.4) | 114 (49.4) | 194 (91.1) | 49 (71.0) |
| Comorbidity: |  |  |  |  |  |  |  |  |  |  |
| Cerebrovascular disease | 71 (24.1) | 361 (20.1) | 91 (21.3) | 48 (14.4) | 52 (17.7) | 97 (19.7) | 32 (23.2) | 29 (12.6) | 60 (28.2) | 22 (31.9) |
| Malignancy | 97 (32.9) | 391 (21.8) | 133 (31.1) | 84 (25.2) | 91 (31.1) | 120 (24.3) | 28 (20.3) | 74 (32.0) | 68 (31.9) | 18 (26.1) |
| Renal disease | 22 (7.5) | 235 (13.1) | 63 (14.8) | 35 (10.5) | 40 (13.7) | 68 (13.8) | 56 (40.6) | 34 (14.7) | 30 (14.1) | 1–9^e^ |
| Congestive heart failure | 115 (39.0) | 651 (36.3) | 213 (49.9) | 137 (41.1) | 173 (59.0) | 228 (46.2) | 75 (54.3) | 103 (44.6) | 81 (38.0) | 27 (39.1) |
| Liver disease | 63 (21.4) | 493 (27.5) | 132 (30.9) | 98 (29.4) | 108 (36.9) | 146 (29.6) | 27 (19.6) | 48 (20.8) | 56 (26.3) | 17 (24.6) |
| Diabetes mellitus | 157 (53.2) | 1,013 (56.4) | 276 (64.6) | 210 (63.1) | 162 (55.3) | 275 (55.8) | 104 (75.4) | 115 (49.8) | 100 (46.9) | 36 (52.2) |
| LTOT before COVID-19 diagnosis | 105 (35.6) | 79 (4.4) | 23 (5.4) | 23 (6.9) | 26 (8.9) | 26 (5.3) | 1–9^e^ | 10 (4.3) | 14 (6.6) | 1–9^e^ |
| COVID-19 treatment: |  |  |  |  |  |  |  |  |  |  |
| Corticosteroids^b^ | 142 (48.1) | 739 (41.2) | 157 (36.8) | 152 (45.6) | 109 (37.2) | 168 (34.1) | 67 (48.6) | 83 (35.9) | 72 (33.8) | 31 (44.9) |
| Steroid pulse^c^ | 65 (22.0) | 140 (7.8) | 30 (7.0) | 33 (9.9) | 15 (5.1) | 21 (4.3) | 22 (15.9) | 1–9^e^ | 16 (7.5) | 1–9^e^ |
| Tocilizumab | 1–9^e^ | 123 (6.9) | 15 (3.5) | 15 (4.5) | 11 (3.8) | 17 (3.4) | 1–9^e^ | 1–9^e^ | 1–9^e^ | 1–9^e^ |
| Baricitinib | 1–9^e^ | 62 (3.5) | 1–9^e^ | 1–9^e^ | 1–9^e^ | 10 (2.0) | 1–9^e^ | 1–9^e^ | 1–9^e^ | 0 |
| Heparin | 46 (15.6) | 253 (14.1) | 54 (12.6) | 38 (11.4) | 40 (13.7) | 51 (10.3) | 25 (18.1) | 27 (11.7) | 18 (8.5) | 1–9^e^ |
| Respiratory support care: |  |  |  |  |  |  |  |  |  |  |
| Oxygen therapy | 206 (69.8) | 658 (36.7) | 139 (32.6) | 103 (30.9) | 97 (33.1) | 144 (29.2) | 62 (44.9) | 77 (33.3) | 99 (46.5) | 28 (40.6) |
| High-flow nasal cannula | 27 (9.2) | 90 (5.0) | 19 (4.4) | 16 (4.8) | 12 (4.1) | 17 (3.4) | 11 (8.0) | 1–9^e^ | 12 (5.6) | 1–9^e^ |
| Mechanical ventilation | 28 (9.5) | 94 (5.2) | 21 (4.9) | 14 (4.2) | 10 (3.4) | 16 (3.2) | 1–9^e^ | 11 (4.8) | 10 (4.7) | 1–9^e^ |
| ECMO | 0 | 1–9^e^ | 1–9^e^ | 0 | 0 | 0 | 0 | 0 | 0 | 0 |
| 60-day mortality | 64 (21.7) | 177 (9.9) | 34 (8.0) | 30 (9.0) | 26 (8.9) | 33 (6.7) | 22 (15.9) | 12 (5.2) | 42 (19.7) | 1–9^e^ |

Data are presented as median age category or number (%)

^a^ Wild-type-predominant wave, January 01, 2020–April, 2021; alpha-predominant wave, April 19, 2021–July 18, 2021; delta-predominant wave, July 19, 2021–August 31, 2021.

^b^ Corticosteroids newly administered within 60 days of COVID-19 diagnosis or corticosteroid dosage increased within 60 days of COVID-19 diagnosis in patients using corticosteroids prior to COVID-19 diagnosis.

^c^ Corticosteroid use equivalent to 500 mg or more of methylprednisolone at least once within 60 days of COVID-19 diagnosis.

^d^ Median age category.

^e^ JMHLW guidelines for the use of claims data require that variables fewer than 10 must not be published. Because the 20–49 age categories contained fewer than 10 variables, they were combined with the 50–64 age category and reported as the 20–64 age category.

ECMO, Extracorporeal membrane oxygenation; IPF, idiopathic pulmonary fibrosis; JMHLW, Japan Ministry of Health, Labor, and Welfare; MPA-ILD, microscopic polyangiitis-associated interstitial lung disease; PM/DM-ILD, polymyositis/dermatomyositis-associated interstitial lung disease; RA-ILD, rheumatoid arthritis-associated interstitial lung disease; SjS-ILD, Sjogren syndrome-associated interstitial lung disease; SLE-ILD, systemic lupus erythematosus-associated interstitial lung disease; SSc-ILD, systemic sclerosis-associated interstitial lung disease

**Table S4. Characteristics of COVID-19 patients without preexisting interstitial lung disease by wave.**

|  |  | **Waves^a^** |  |  | **Wild-type vs. Alpha^b^** | **Alpha vs. Delta^b^** | **Wild-type vs. Delta^b^** |
| --- | --- | --- | --- | --- | --- | --- | --- |
|  | Wild-type  n = 361,542 | Alpha  n = 195,284 | Delta  n = 373,599 |  | Difference  % (95% CI) | Difference  % (95% CI) | Difference  % (95% CI) |
| Age, years: | 45–49^e^ | 45–49^e^ | 35–39^e^ |  |  |  |  |
| 20–49 | 186,792 (51.7) | 111,111 (56.9) | 278,724 (74.6) |  | 5.2 (5.0 to 5.5) | 17.7 (17.4 to 18.0) | 22.9 (22.7 to 23.2) |
| 50–64 | 72,345 (20.0) | 40,921 (21.0) | 70,573 (18.9) |  | 0.9 (0.7 to 1.2) | −2.1 (−2.3 to −1.8) | −1.1 (−1.3 to −0.9) |
| 65–79 | 58,818 (16.3) | 26,783 (13.7) | 15,897 (4.3) |  | −2.6 (−2.7 to −2.4) | −9.5 (−9.6 to −9.3) | −12.0 (−12.2 to −11.9) |
| 80+ | 43,587 (12.1) | 16,469 (8.4) | 8,405 (2.2) |  | −3.6 (−3.8 to −3.5) | −6.2 (−6.3 to −6.1) | −9.8 (−9.9 to −9.7) |
| Sex, male | 192,677 (53.3) | 105,485 (54.0) | 208,692 (55.9) |  | 0.7 (0.4 to 1.0) | 1.8 (1.6 to 2.1) | 2.6 (2.3 to 2.8) |
| Comorbidity: |  |  |  |  |  |  |  |
| Cerebrovascular disease | 30,705 (8.5) | 12,517 (6.4) | 9,082 (2.4) |  | −2.1 (−2.2 to −1.9) | −4.0 (−4.1 to −3.9) | −6.1 (−6.2 to −6.0) |
| Malignancy | 22,897 (6.3) | 9,554 (4.9) | 8,049 (2.2) |  | −1.4 (−1.6 to −1.3) | −2.7 (−2.8 to −2.6) | −4.2 (−4.3 to −4.1) |
| Renal disease | 11,443 (3.2) | 4,717 (2.4) | 3,426 (0.9) |  | −0.8 (−0.8 to −0.7) | −1.5 (−1.6 to −1.4) | −2.2 (−2.3 to −2.2) |
| Congestive heart failure | 31,903 (8.8) | 13,035 (6.7) | 9,795 (2.6) |  | −2.1 (−2.3 to −2.0) | −4.1 (−4.2 to −3.9) | −6.2 (−6.3 to −6.1) |
| Liver disease | 36,939 (10.2) | 17,892 (9.2) | 20,807 (5.6) |  | −1.1 (−1.2 to −0.9) | −3.6 (−3.7 to −3.4) | −4.6 (−4.8 to −4.5) |
| Diabetes mellitus | 56,956 (15.8) | 26,203 (13.4) | 24,981 (6.7) |  | −2.3 (−2.5 to −2.1) | −6.7 (−6.9 to −6.6) | −9.1 (−9.2 to −8.9) |
| LTOT before COVID-19 diagnosis | 889 (0.2) | 332 (0.2) | 205 (0.1) |  | −0.1 (−0.1 to −0.1) | −0.1 (−0.1 to −0.1) | −0.2 (−0.2 to −0.2) |
| COVID-19 treatment: |  |  |  |  |  |  |  |
| Corticosteroids^c^ | 55,375 (15.3) | 39,619 (20.3) | 39,145 (10.5) |  | 5.0 (4.8 to 5.2) | −9.8 (−10.0 to −9.6) | −4.8 (−5.0 to −4.7) |
| Steroid pulse^d^ | 5,070 (1.4) | 3,857 (2.0) | 2,730 (0.7) |  | 0.6 (0.5 to 0.6) | −1.2 (−1.3 to −1.2) | −0.7 (−0.7 to −0.6) |
| Tocilizumab | 2,939 (0.8) | 2,447 (1.3) | 2,061 (0.6) |  | 0.4 (0.4 to 0.5) | −0.7 (−0.8 to −0.6) | −0.3 (−0.3 to −0.2) |
| Baricitinib | 209 (0.1) | 5,330 (2.7) | 6,239 (1.7) |  | 2.7 (2.6 to 2.7) | −1.1 (−1.1 to −1.0) | 1.6 (1.6 to 1.7) |
| Heparin | 18,559 (5.1) | 10,493 (5.4) | 7,448 (2.0) |  | 0.2 (0.1 to 0.4) | −3.4 (−3.5 to −3.3) | −3.1 (−3.2 to −3.1) |
| Respiratory support care: |  |  |  |  |  |  |  |
| Oxygen therapy | 48,816 (13.5) | 27,459 (14.1) | 21,711 (5.8) |  | 0.6 (0.4 to 0.7) | −8.3 (−8.4 to −8.1) | −7.7 (−7.8 to −7.6) |
| High-flow nasal cannula | 3,758 (1.0) | 3,624 (1.9) | 3,019 (0.8) |  | 0.8 (0.7 to 0.9) | −1.0 (−1.1 to −1.0) | −0.2 (−0.3 to −0.2) |
| Mechanical ventilation | 7,632 (2.1) | 3,402 (1.7) | 2,062 (0.6) |  | −0.4 (−0.4 to −0.3) | −1.2 (−1.3 to −1.1) | −1.6 (−1.6 to −1.5) |
| 60-day mortality | 10,078 (2.8) | 3,996 (2.0) | 1,591 (0.4) |  | −0.7 (−0.8 to −0.7) | −1.6 (−1.7 to −1.6) | −2.4 (−2.4 to −2.3) |

Data are presented as median age category or percent.

^a^ Wild-type-predominant wave, January 01, 2020–April 18, 2021; alpha-predominant wave, April 19, 2021–July 18, 2021; delta-predominant wave, July 19, 2021–August 31, 2021.

^b^ Earlier wave was used as reference.

^c^ Corticosteroids newly administered within 60 days of COVID-19 diagnosis or corticosteroid dosage increased within 60 days of COVID-19 diagnosis in patients using corticosteroids prior to COVID-19 diagnosis.

^d^ Corticosteroid use equivalent to 500mg or more of methylprednisolone at least once within 60 days of COVID-19 diagnosis.

^e^ Median age category.

CI, confidence interval; ILD, interstitial lung disease; LTOT, long term oxygen therapy

**Table S5. Change of mortality by etiology in COVID-19 patients with preexisting interstitial lung disease.**

|  |  | **Waves^a^** |  |  | **Wild-type vs. Alpha^b^** | **Alpha vs. Delta^b^** | **Wild-type vs. Delta^b^** |
| --- | --- | --- | --- | --- | --- | --- | --- |
|  | Wild-type  mortality, % | Alpha  mortality, % | Delta  mortality, % |  | Difference  % (95% CI) | Difference  % (95% CI) | Difference  % (95% CI) |
| Idiopathic pulmonary fibrosis | 23.6 | 19.0 | 16.7 |  | −4.6 (−16.3 to 7.1) | −2.3 (−17.4 to 12.8) | −6.9 (−19.7 to 5.8) |
| RA-ILD | 12.0 | 9.9 | 4.4 |  | −2.0 (−5.6 to 1.5) | −5.6 (−9.1 to −2.0) | −7.6 (−10.5 to −4.7) |
| SLE-ILD | 11.0 | 8.4 | 1.8 |  | −2.5 (−9.5 to 4.4) | −6.7 (−12.7 to −0.6) | −9.2 (−14.0 to −4.4) |
| PM/DM-ILD | 10.0 | 9.2 | 6.0 |  | −0.8 (−8.6 to 7.0) | −3.2 (−11.9 to 5.4) | −4.0 (−11.1 to 3.1) |
| SSc-ILD | 12.0 | 5.8 | 2.0 |  | −6.2 (−13.5 to 1.1) | −3.8 (−10.5 to 3.0) | −10.0 (−16.2 to −3.7) |
| SjS-ILD | 6.0 | 10.7 | 4.3 |  | 4.7 (−1.7 to 11.1) | −6.4 (−13.2 to 0.5) | −1.7 (−6.4 to 3.0) |
| MPA-ILD | 17.9 | 17.6 | 7.7 |  | −0.3 (−15.7 to 15.1) | −10.0 (−26.4 to 6.5) | −10.3 (−23.6 to 3.1) |
| Pulmonary sarcoidosis | 4.5 | 10.5 | 1.6 |  | 6.1 (−2.8 to 14.9) | −8.9 (−17.5 to −0.4) | −2.9 (−7.8 to 2.1) |
| Pneumoconiosis | 23.1 | 15.7 | 10.7 |  | −7.4 (−19.7 to 4.8) | −5.0 (−20.2 to 10.2) | −12.4 (−25.9 to 1.1) |
| Hypersensitivity pneumonitis | 13.3 | 15.4 | 9.1 |  | 2.1 (−19.9 to 24.0) | −6.3 (−32.2 to 19.7) | −4.2 (−23.9 to 15.4) |

^a^ Wild-type-predominant wave, January 01, 2020–April 18, 2021; alpha-predominant wave, April 19, 2021–July 18, 2021; delta-predominant wave, July 19, 2021–August 31, 2021.

^b^ Earlier wave was used as reference.

CI, confidence interval; ILD, interstitial lung disease; JMHLW, Japan Ministry of Health, Labor, and Welfare; MPA-ILD, microscopic polyangiitis-associated interstitial lung disease; PM/DM-ILD, polymyositis/dermatomyositis-associated interstitial lung disease; RA-ILD, rheumatoid arthritis-associated interstitial lung disease; SjS-ILD, Sjogren syndrome-associated interstitial lung disease; SLE-ILD, systemic lupus erythematosus-associated interstitial lung disease; SSc-ILD, systemic sclerosis-associated interstitial lung disease
